# European and Developing Countries Clinical Trials Partnership (EDCTP): the path towards a true partnership

**DOI:** 10.1186/1471-2458-9-249

**Published:** 2009-07-20

**Authors:** Mecky I Matee, Christine Manyando, Peter M Ndumbe, Tumani Corrah, Walter G Jaoko, Andrew Y Kitua, Herman PA Ambene, Mathieu Ndounga, Lynn Zijenah, David Ofori-Adjei, Simon Agwale, Steven Shongwe, Thomas Nyirenda, Michael Makanga

**Affiliations:** 1 Department of Microbiology and Immunology, Muhimbili University of Health and Allied Sciences, P.O. Box 65001, Dar es Salaam, Tanzania; 2 Tropical Disease Reseach Centre, P.O. Box 71769, Ndola, Zambia; 3 Faculty of Health Sciences, University of Buea, BP 63 Buea, Cameroon; 4 Medical Research Council, Fajara, P.O. Box 273, Banjul, The Gambia; 5 Department of Medical Microbiology, University of Nairobi, P.O. Box 19676 Nairobi, Kenya; 6 National Institute for Medical Research, P.O. Box 9653, Dar es Salaam, Tanzania; 7 Organization de Coordination Pour la Lutte Contre Les Endemies en Afrique Centrale (OCEAC), BP 288, Yaoundé, Cameroon; 8 Centre d' etudes sur resources vegetales, BP 1249, Brazzaville, Congo; 9 Department of Immunology, University of Zimbabwe Medical School, P.O. Box A178 Avondale, Harare, Zimbabwe; 10 Noguchi Memorial Institute for Medical Research College of Health Science, University of Ghana, P. O. Box LG 581 Accra, Ghana; 11 Innovative Biotech, Abdu Abubakar Street GRA, P.O. Box 30 Keffi, Nasarawa State, Nigeria; 12 WHO Regional Office for East, Central and Sourthen Africa, Arusha, Tanzania; 13 EDCTP Africa Office, MRC P.O. Box 19070, Francie van Zyl Street Tygerberg, Cape Town, South Africa

## Abstract

**Background:**

European and Developing Countries Clinical Trials Partnership (EDCTP) was founded in 2003 by the European Parliament and Council. It is a partnership of 14 European Union (EU) member states, Norway, Switzerland, and Developing Countries, formed to fund acceleration of new clinical trial interventions to fight the human immunodeficiency virus and acquired immune deficiency syndrome (HIV/AIDS), malaria and tuberculosis (TB) in the sub-Saharan African region. EDCTP seeks to be synergistic with other funding bodies supporting research on these diseases.

**Methods:**

EDCTP promotes collaborative research supported by multiple funding agencies and harnesses networking expertise across different African and European countries. EDCTP is different from other similar initiatives. The organisation of EDCTP blends important aspects of partnership that includes ownership, sustainability and responds to demand-driven research. The Developing Countries Coordinating Committee (DCCC); a team of independent scientists and representatives of regional health bodies from sub-Saharan Africa provides advice to the partnership. Thus EDCTP reflects a true partnership and the active involvement and contribution of these African scientists ensures joint ownership of the EDCTP programme with European counterparts.

**Results:**

The following have been the major achievements of the EDCTP initiative since its formation in 2003; i) increase in the number of participating African countries from two to 26 in 2008 ii) the cumulative amount of funds spent on EDCTP projects has reached € 150 m, iii) the cumulative number of clinical trials approved has reached 40 and iv) there has been a significant increase number and diversity in capacity building activities.

**Conclusion:**

While we recognise that EDCTP faced enormous challenges in its first few years of existence, the strong involvement of African scientists and its new initiatives such as unconditional funding to regional networks of excellence in sub-Saharan Africa is envisaged to lead to a sustainable programme. Current data shows that the number of projects supported by EDCTP is increasing. DCCC proposes that this success story of true partnership should be used as model by partners involved in the fight against other infectious diseases of public health importance in the region.

## Background

Tuberculosis, human immunodeficiency virus (HIV) and malaria cross paths in sub-Saharan Africa, the epicentre of the three infections. Although HIV/AIDS, tuberculosis (TB) and malaria are three treatable and preventable diseases, they are having a devastating impact in the world's poorest countries. Sub-Saharan Africa has just over 10% of the world's population but accounts for 90% of malaria deaths, two-thirds of all people living with HIV, and nearly one-third of all TB cases [1]. The human impact of these three diseases is undeniable, but their social and economic impacts are also severe. Thus investments in the fight against HIV/AIDS, tuberculosis and malaria will lead to very significant improvements in health and lives of people across sub-Saharan Africa.

Global resources devoted to fighting the three diseases have been rapidly scaled up in recent years by various initiatives or programs including the World Health Organization (WHO), Tropical Diseases Research (TDR), Foundation for Innovative New Diagnostics (FIND), the US Centers for Disease Control and Prevention (CDC), National Institute of Health (NIH), European Union (EU), Wellcome Trust (WT), Multilateral Initiative on Malaria in Africa (MIM), to mention a few. These initiatives have achieved significant results in the areas of capacity building, networking, and development of new tools for intervention. One such initiative was the formation in 2003, of the European and Developing Countries Clinical Trials Partnership (EDCTP), a partnership of 14 European Union (EU) member states, Norway and Developing Countries, with a particular focus on sub-Saharan African states. EDCTP was formed to develop new clinical trial interventions to fight the human immunodeficiency virus/acquired immune deficiency syndrome (HIV/AIDS), malaria and tuberculosis (TB) in the sub-Saharan region. Switzerland joined the partnership in 2006.

EDCTP has sought to be synergistic with other funding bodies supporting research on these three diseases and promoted collaborative research involving support from multiple funding agencies and harnessing networking expertise across different African and European countries [2]. Formation of EDCTP followed an appeal from African leaders (July 2000) [3] found in the Abuja Declaration (April 2001) [4] that specified the need to find interventions to ensure adequate and effective control of malaria, HIV/AIDS, TB and other related infectious diseases in the African continent. The program was adopted by the European Parliament and the Council in June 2003 [5]; with a 200 million Euros contribution from the European Commission (EC) over 5 years, and expected matching funds from European National Programmes and other parties such as pharmaceutical industries. This agreement is contained in Article 169 of the EC treaty that allows participation of the European Community in EU member states' national research and development programmes [6].

The activities of EDCTP are in seven categories namely: North-North/North-South networking, South-South (intra sub-Saharan) networking, support to clinical trials, support to research capacity building, advocacy and fund raising, management and information management.

The notion of establishing and strengthening of research capacity in developing countries adopted by EDCTP is of prime importance in empowering these countries to find rational and efficient solutions to their health problems through scientific research. It is also known that institutional links with other researchers in the north and south greatly facilitate the process of research strengthening through graduate study programs, technology transfer, 'hands-on' research training in the field, expanded networking with contacts of other partners and continued scientific exchanges in the context of actual research programs [7]. In North-North and North-South networking EDCTP aims to promote and facilitate coordination and pooling of resources among EU states, and to form alliances between European institutions and African partners. Consequently, EDCTP periodically launches joint calls for grants to European National Programmes for North-North networking, capacity building and clinical trials programmes in Africa. Similarly, for South-South networking EDCTP promotes and facilitates creation of networks of African scientists and their European collaborators including links with existing research networks on the three major diseases. EDCTP's mandate is the funding of phases II and III clinical trials although other phases may be included in special circumstances. The emphasis on these phases of trials is to expedite promising health interventions from upstream research to product registration and use by public health systems in needy countries. EDCTP works in line with science and technology strategies of bodies like the EU, the New Economic Partnership for African Development (NEPAD) and the African Union (AU).

There is need to explain what makes EDCTP different from other similar initiatives. We report that in the short history of EDCTP a model of a true partnership has emerged.

### Partnership base

The governance of EDCTP has two pillars namely: the "Partnership" and the "European Economic Interest Group" (EEIG). The "Partnership" comprises the Partnership Board (PB), the main strategic body; network of European National Programs (ENNP); and the Developing Countries Co-coordinating Committee (DCCC). The EEIG provides the legal, financial and operational procedures to receive, dispense, account for funds and execute actions recommended by the PB. The DCCC is a committee of African scientists and representatives of regional health organisations from sub-Saharan Africa that focus on clinical trials in the three major diseases. The importance of DCCC is reflected in the true value of independent scientists from sub-Saharan Africa who advise the partnership in several aspects of its work and is an indication of the African contribution. The DCCC shapes the research agenda according to needs and gaps in the African region. Additional African contribution comes in forms of study sites that have research infrastructure, personnel and leadership.

### Ownership

In line with the Article 169 of the European Treaty, the success of the EDCTP depends on a solid and sustained collaboration of European national programmes to provide and complement the required funding. The active involvement of African scientists and African contribution explained above is also a sign of joint ownership of the EDCTP programme. Although data on financial contribution from African governments, who mostly contribute staff, infrastructure and facilitates participation of consenting study subjects in EDCTP funded projects, has yet to be defined active participation by many African scientists either as DCCC members or grantees is a sign that the EDCTP programme has been embraced in sub-Saharan Africa. The European national programmes and the study sites in Africa are dependent on each other for success of the programme, ensuring a joint ownership.

### Capacity building

The EDCTP capacity building strategy in Africa is unique among others in addressing the 10/90 gap [8], for example. It offers full support only to African scientists, promotes African ownership of projects, receives backing from DCCC who have full sub-Saharan Africa regional representation and receives scientific advice from the Partnership Board half of whose membership is African. DCCC has also recently designed a strategy of unconditional funding to regional networks of excellence in sub-Saharan Africa. These networks will comprise research institutions with complementary research expertise that will provide training and mentorship to less endowed centres in their regions to bring them to levels where they can participate more effectively in multi-centre clinical trials. This will provide a conducive working environment to health research scientists and help to retain them in the four African regions of east, west, centre and south. In addition, the insistence on stronger institutions building capacity in less endowed ones within clinical trials has helped to ensure that there is equitable distribution of funds for capacity building in Africa.

### Sustainability

We recognise that the first few years of EDCTP's existence was full of challenges expected of any newly formed organisation. One of these challenges manifested between 2003 and 2005 when there were rapid changes in management of EDCTP resulting in comments that the management structure of the organization needed to be radically changed and that partnership with other organizations needed to be improved [9]. However taking into consideration the factors stated in the partnership and ownership sections above there is currently some evidence of a sustainable programme.

### Demand-driven research

Combating the three poverty related diseases on which EDCTP focuses is a global emergency and the world needs quick solutions. Inevitably, since its formation, EDCTP has been under pressure to contribute to finding these solutions. Some authors had predicted that the EDCTP will help to overcome the bottleneck of demonstrating a proof of principle for promising vaccine or drug candidates in testing them in early efficacy trials in endemic areas, particularly in sub-Saharan Africa [10]. How long it would have taken for a new organisation to reach this goal was not conceptualised. The advertising of EDCTP with a funding of 200 million Euros from the EC and matching funds from the European member states created a lot of expectations. Currently available data shows that in the past two years the situation has improved. This has brought some discussions on how to include other diseases of poverty and use the EDCTP model to address those health problems.

### New face of EDCTP

**Figure 1:**
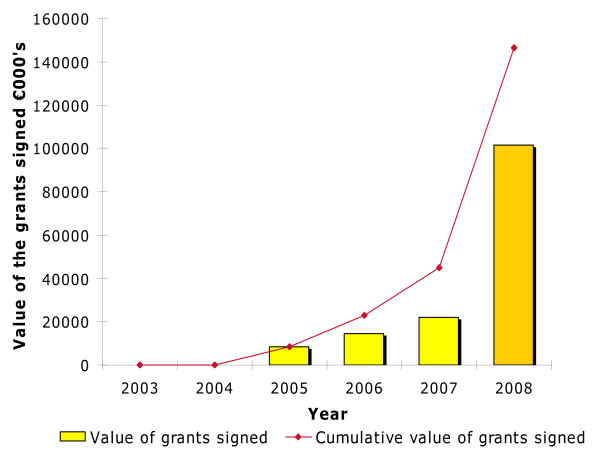
**Amount spent on grants in Euros from 2003 to 2008 project expenditure**.

**Figure 2:**
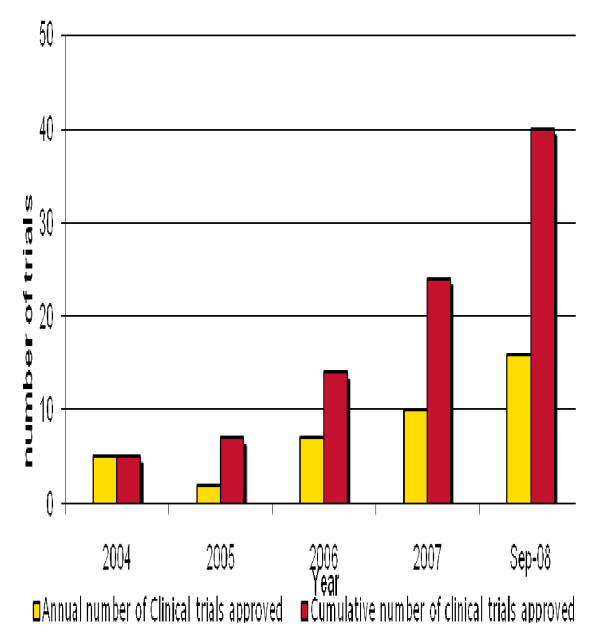
**Number of trials supported with EDCTP grants from 2004 to 2008**.

Since 2005 there have been substantial improvements in the management and awarding of EDCTP grants. By the end of 2007 EDCTP had funded 74 projects in 29 sub-Saharan Africa countries. A summary of these projects are available in EDCTP annual reports [11,12] and the EDCTP website . Some key performance indicators that are transparent monitoring tools have been used to show significant progress. Figure 1 shows the amount of funds that have been spent on all EDCTP projects since 2003. A substantial increase in the funded projects occurred between 2005 and 2006 as illustrated in Figure 2.

**Figure 3:**
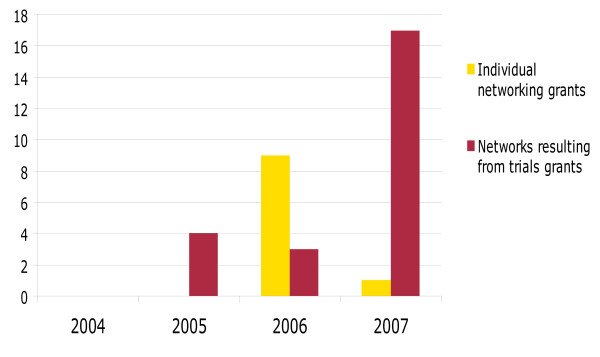
**Number of integrated projects and networking grants**.

Grants to networking projects and clinical trials have resulted in formation of new networks or support to on-going ones as illustrated in Figure 3. It is envisaged that this will increase further in 2008 due to unconditional funding to regional networks of excellence soon to commence. Please insert a table of list of projects.

**Figure 4:**
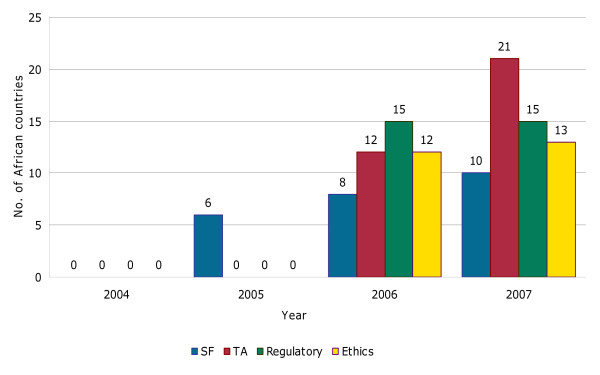
**Number of capacity building (senior fellowship, SF; Training award, TA; regulatory training and ethics strengthening) activities granted EDCTP funding from 2005 to 2007**.

**Figure 5:**
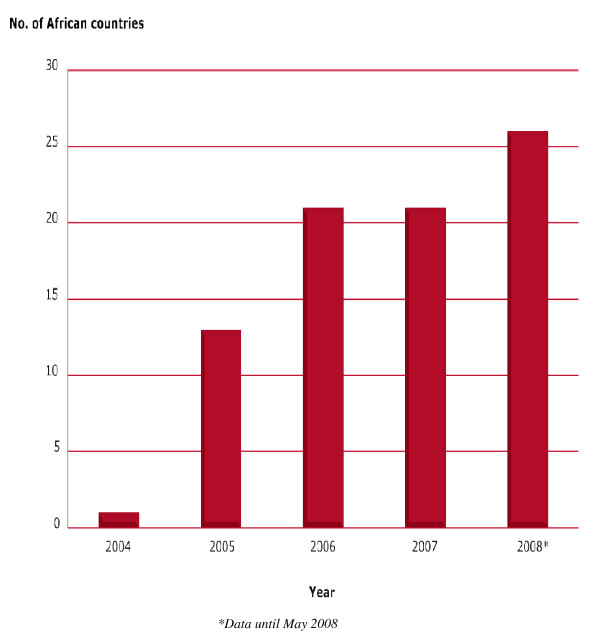
**Number of African countries involved in EDCTP projects from 2004 to 2008**.

After 2006 the DCCC and other EDCTP constituencies have insisted on demonstration of African leadership and ownership in the EDCTP programme. Capacity building programmes have been tremendously supported as shown in Figure 4. The number of African countries involved in EDCTP funded projects keep increasing as illustrated in Figure 5. The next challenge is shaping the future direction of the organisation.

### The future of EDCTP

We have this far argued that EDCTP has a good future as long as it receives strong support from both African and European member states in the partnership. As DCCC we propose that the future of EDCTP should include the following elements:

1) Stronger investment for research in the three major diseases of HIV, TB and malaria

2) Stronger African commitment and leadership

3) Consideration to put European member state funding into a common pot which currently is not the case

4) Inclusion of basic sciences (discovery research) and phases I and IV studies in the mandate of EDCTP; consolidated in regional networks of excellence

5) Expansion of disease coverage beyond the current three poverty related diseases to other neglected ones

6) Support for standardisation of methods for evaluation of effectiveness of clinical interventions e.g. biomarkers and correlates of protection, cure etc

7) Funding drug discovery of traditional medicinal plants

8) Continued support for regulatory and ethics activities

9) Negotiations with pharmaceutical companies for third party funding of product research

Some of these points are also contained in the recent EDCTP independent external evaluation [13]. We believe that in implementing these recommendations over and above the current programme, EDCTP will become an even stronger partner in fighting poverty by improving health of populations and continue to serve as a best example of partnerships between developed and least developed countries in combating regional public health problems.

## Conclusion

Despite facing enormous challenges in its first few years of existence, EDCTP has succeeded in getting strong involvement of African scientists and, through its new initiatives, such as unconditional funding to regional networks of excellence in sub-Saharan Africa, it is envisaged to lead to a sustainable programme. DCCC proposes that this success story of true partnership should be used as model by partners involved in the fight against other infectious diseases of public health importance in the region.

## Competing interests

NT and MM work in the African office of the EDCTP. The other authors declare that they have no competing interests.

## Authors' contributions

DCCC members wrote the paper and edited the first draft and provided necessary background documents.

## Pre-publication history

The pre-publication history for this paper can be accessed here:



## References

[B1] Anonymous Universal Access to HIV/AIDS, Tuberculosis and Malaria Services a United Africa by 2010. Special summit of African union on HIV/AIDS, tuberculosis and malaria (ATM) Abuja, Nigeria. 2 – 4 May, 2006. http://www.aidsuganda.org/sero/African%20Union.pdf.

[B2] Olliaro P, Smith PG (2004). The European and Developing countries clinical trials partnership. J HIV Ther.

[B3] The African Development Forum 2000 Report Leadership at all levels to overcome HIV/AIDS.

[B4] The Abuja declaration on HIV/AIDS, tuberculosis, malaria and other related infectious diseases AIDS the greatest leadership challenge.

[B5] (2003). Decision No 1209/2003/EC of 16 June 2003 OJ L 169, 97.

[B6] Commission of the European Communities (2004). Second progress report on EC Programme for Action: Accelerated action of HIV/AIDS, malaria and tuberculosis in the context of poverty reduction. Brussels SEC (2004) 1326.

[B7] Lansang MA, Olveda RO (1994). Institutional links: strategic bridges for research capacity strengthening. Acta Trop.

[B8] Global Forum for Health Research (2004). The 10/90 Report on Health Research 2003–2004. Geneva, Switzerland.

[B9] The PLoS Medicine Editors (2004). A new vision for Clinical Trials in Africa: A promising European funding body is stumbling over the details. PLoS Medicine.

[B10] Medaglini D, Hoeveler A (2003). The European research effort for HIV/AIDS, malaria and tuberculosis. Vaccine.

[B11] Europe Developing Countries Clinical Trials Partnership Annual report 2005. http://www.edctp.org.

[B12] Europe Developing Countries Clinical Trials Partnership Annual report 2006. http://www.edctp.org.

[B13] Europe Developing Countries Clinical Trials Partnership Independent External Review. IER/12 July 2007. http://www.edctp.org.

